# A reference-grade genome identifies salt-tolerance genes from the salt-secreting mangrove species *Avicennia marina*

**DOI:** 10.1038/s42003-021-02384-8

**Published:** 2021-07-08

**Authors:** Purushothaman Natarajan, Ashok Kumar Murugesan, Ganesan Govindan, Ayyaru Gopalakrishnan, Ravichandiran Kumar, Purushothaman Duraisamy, Raju Balaji, Tanuja, Puhan Sushree Shyamli, Ajay K. Parida, Madasamy Parani

**Affiliations:** 1 Genomics Laboratory, Department of Genetic Engineering, SRM Institute of Science and Technology, Kattankulathur, Tamil Nadu India; 2 Centre of Advanced Study in Marine Biology, Faculty of Marine Sciences, Annamalai University, Parangipettai, Tamil Nadu India; 3 Institute of Life Sciences, NALCO Square, Bhubaneswar, India

**Keywords:** Genomics, Plant sciences

## Abstract

Water scarcity and salinity are major challenges facing agriculture today, which can be addressed by engineering plants to grow in the boundless seawater. Understanding the mangrove plants at the molecular level will be necessary for developing such highly salt-tolerant agricultural crops. With this objective, we sequenced the genome of a salt-secreting and extraordinarily salt-tolerant mangrove species, *Avicennia marina*, that grows optimally in 75% seawater and tolerates >250% seawater. Our reference-grade ~457 Mb genome contains 31 scaffolds corresponding to its chromosomes. We identified 31,477 protein-coding genes and a salinome consisting of 3246 salinity-responsive genes and homologs of 614 experimentally validated salinity tolerance genes. The salinome provides a strong foundation to understand the molecular mechanisms of salinity tolerance in plants and breeding crops suitable for seawater farming.

## Abstract (web summary)

Natarajan et al. present a reference-grade genome for an ecologically important species of salt-tolerant mangrove tree. They identify a large number of salinity-responsive genes that may be involved in salt tolerance.

## Introduction

**Fig. 1:**
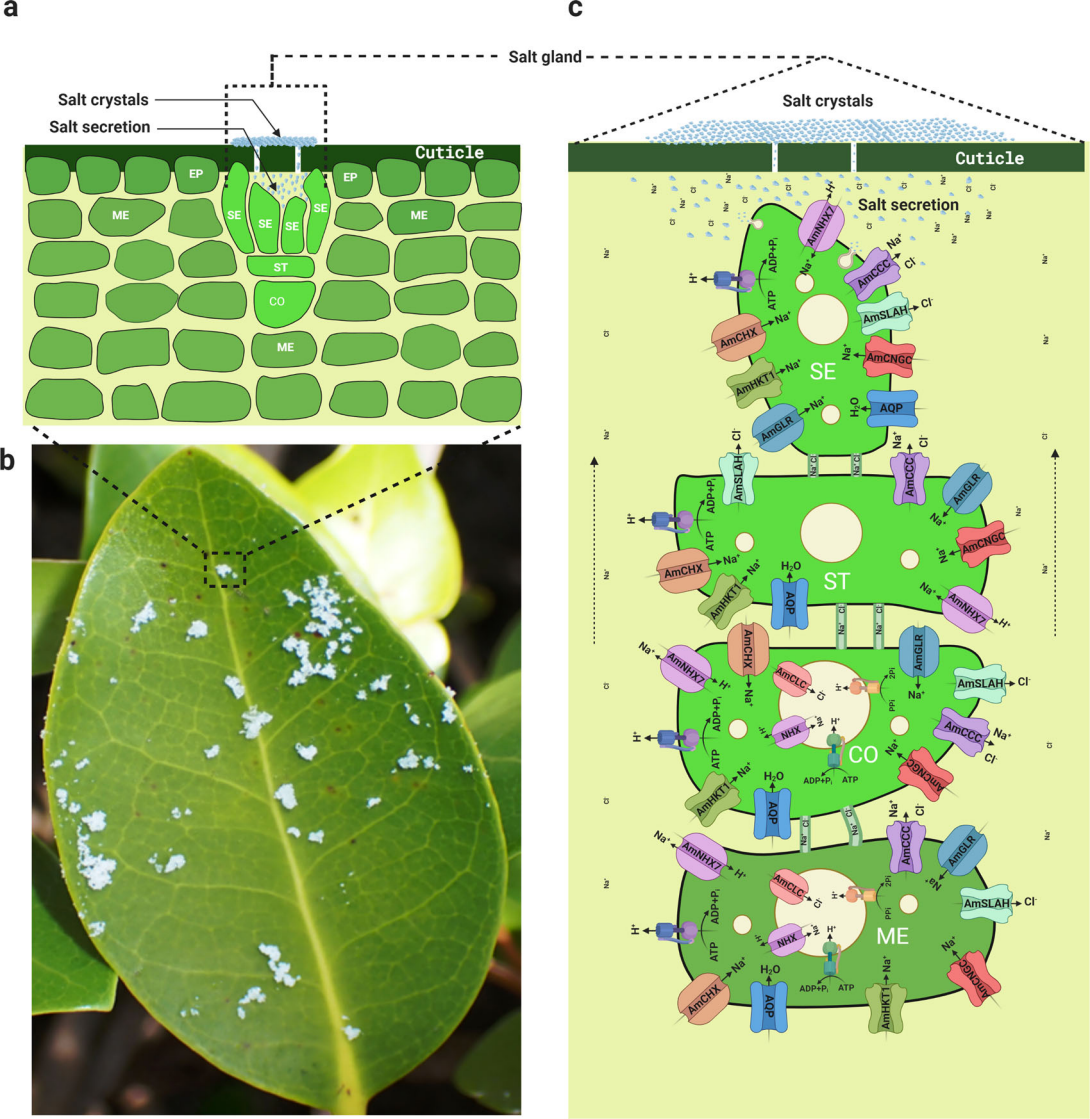
Secretion of salts through the salt glands of *Avicennia*. **a** Schematic of a salt gland showing the mesophyll cells (ME), collecting cells (CO), stalk cell (ST), secreting cells (SE), and epidermal cells (EP). **b** Salts secreted by the salt glands and crystalized on the leaf surface. **c** Hypothetical mechanism of salt secretion through the salt glands involving ion channels, aquaporins, plasmodesmata, and vesicles. NHX sodium/hydrogen exchanger, CHX cation:proton antiporters, CCC cation-chloride cotransporter, SLAH slow anion channel-associated homologs, CNGC cyclic nucleotide-gated cation channel, GLR glutamate-activated channels, AQP aquaporin, CLC chloride channel, HKT1 high-affinity K+ transporter 1.

The world population is expected to increase by more than 20 percent to ~9 billion by 2050^1^. With 2 billion people already not having sufficient food^2^, crop production needs to increase significantly to feed the ever-increasing global population. Availability of water is a significant challenge to crop production in dryland areas, which accounts for ~40 percent of the world’s total land area^3^. Another water-related problem, salinity, is prevalent in ~900 million hectares, and it is estimated to cause an annual loss of 27 billion USD^4–6^. Though water makes up 71% of the earth’s surface, 96.5% is saline seawater unsuitable for growing plants. However, an exception to this is the mangrove plants that thrive in seawater and are central to the most productive tropical mangrove ecosystem^7^. Evidence suggests that mangrove plants evolved during the Late Cretaceous and early Tertiary period or Paleocene–Eocene epoch from independent terrestrial plant lineages exposed to the seawater^8^. While the mangrove plant’s remarkable adaptions to an otherwise harsh ecological setting have been studied^9,10^, our understanding is far from complete. Specialized mangrove roots with hydrophobic barriers and ultrafiltration mechanisms exclude 80 to 97% of salts^11–14^; nevertheless, continuous exposure to seawater leads to the absorption of a significant quantity of salts. To avoid the accumulation of excess salts, the leaves of some mangroves have specialized glands (Fig. 1) that excrete about 40% of the absorbed salts^12,15^. Osmotic regulation, ion sequestration, antioxidant enzymes, and compatible solutes protect the tissues from the detrimental effects of the salt that remains inside the plant system after exclusion and secretion^16,17^.

The advances in genome sequencing and assembly technologies have enhanced our understanding of many plants and animals. While gene expression analysis and whole-genome sequencing studies are beginning to provide a molecular understanding of the mangrove plants^10,18–25^, reference-grade genome assembly, which is essential to carry out a comprehensive study on salinity tolerance genes at the whole-genome level, is not available for any mangrove species. In this study, we sequenced the genome and analyzed the salinity tolerance genes of *Avicennia marina*, an extremely salt-tolerant mangrove species with salt glands^12,26^. We report a 457 Mb reference-grade genome that contains 31,477 protein-coding genes. Further, we found that about 12% (3860 genes) of the *A. marina* genes constitute the salinome, a set of genes associated with salinity tolerance.

## Results

### Genome sequencing and assembly

**Fig. 2:**
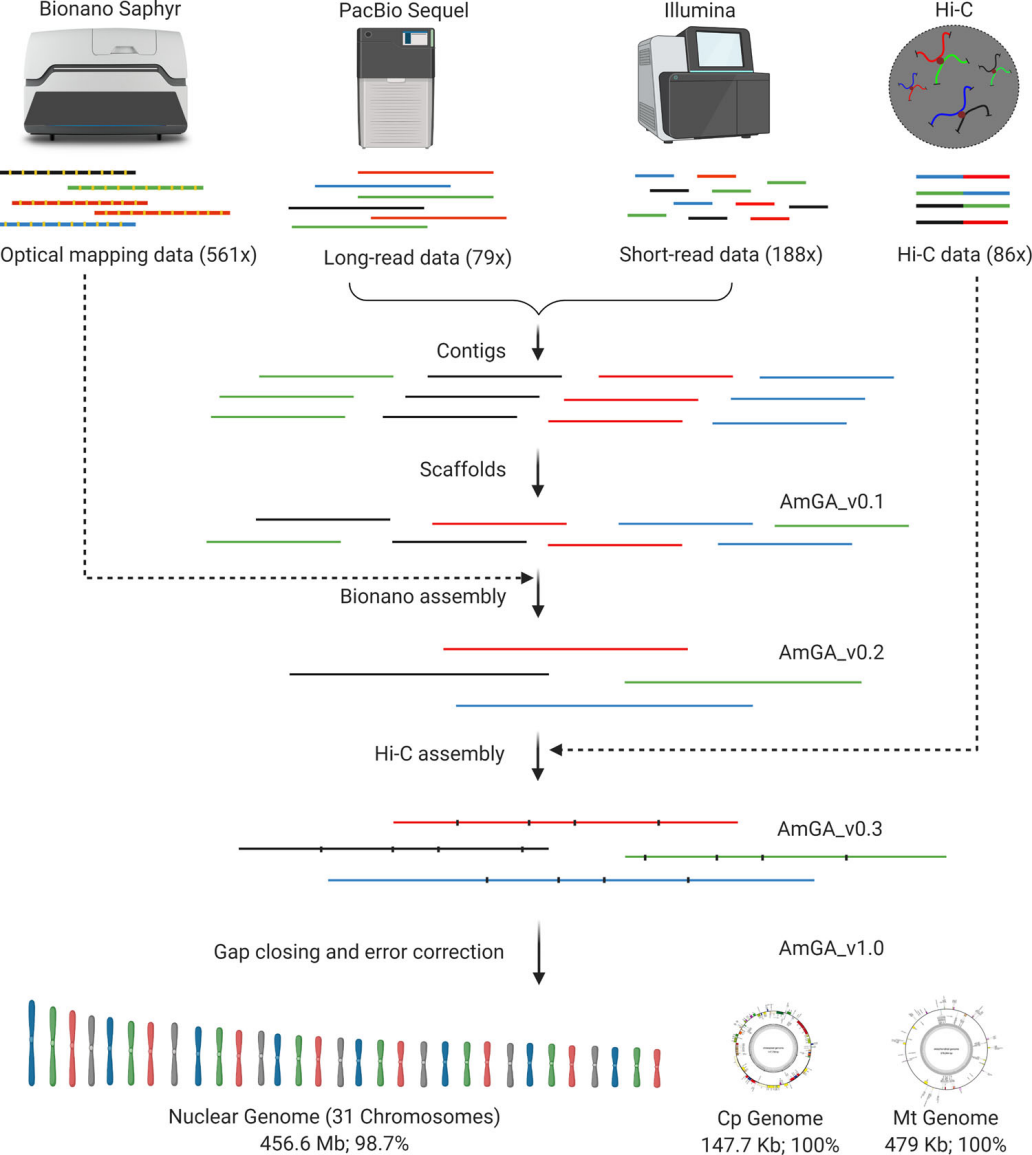
Schematic of the de novo assembly of *A. marina* genome. Long-read and short-read data from PacBio Sequel and Illumina, respectively, were subjected to hybrid assembly to build contigs that were combined to build scaffolds. Super-scaffolds were built by combining the scaffolds from Bionano assembly and those from the previous step. Chromosome-scale assembly was built using the Hi-C chromosome conformation capture data, and the assembly was further refined by gap closing and error correction. Sequence data from a scaffold that contained the organellar genomes were re-assembled to build full-length circular chloroplast and mitochondrial genomes.

**Fig. 3:**
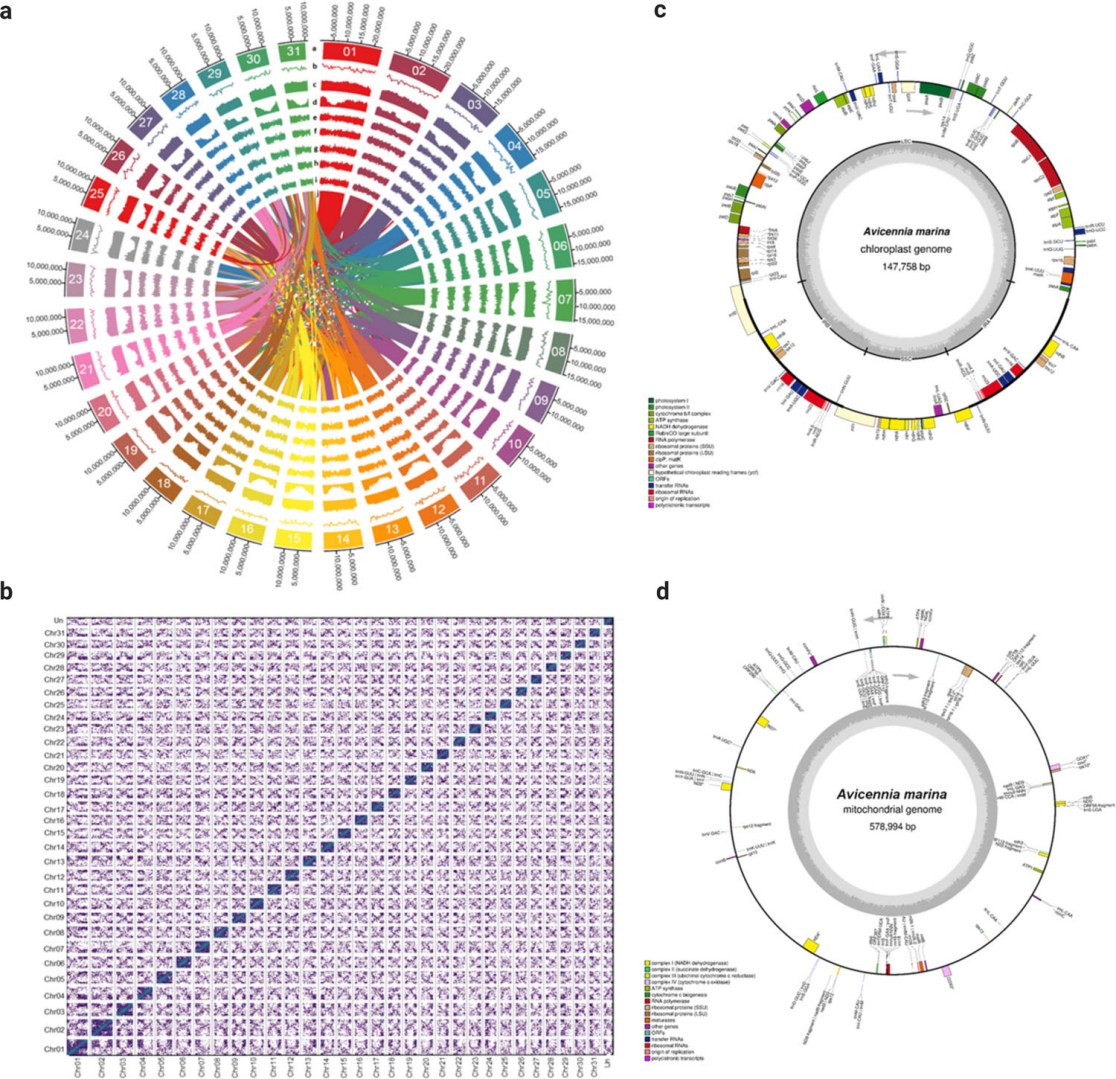
*Avicennia marina* genome features. **a** Circos plot of the genome representing 31 chromosomes (a), distribution of the GC content (b), repeat content (c) and gene density (d) calculated in 0.5 Mb window size, expression profile of the genes from leaves, roots, flowers, pneumatophores, and seeds, respectively (e–i), and synteny blocks from intrachromosomal links (j). The bandwidth is proportional to synteny block size. Source data are provided as a Source Data file. Circular representation of chloroplast genome. **b** Hi-C interaction matrix for *A. marina* genome assembly with 32 clusters. **c** Circular representation of chloroplast genome. **d** Circular representation of the mitochondrial genome.

We estimated the haploid genome size of *A. marina* to be 462.7 Mb with 0.36% heterozygosity based on k-mer analysis (Supplementary Fig. 1). We first obtained 36.6 Gb (~79×) of long-read Pacbio sequence data, with the N50 size of 13.23 kb. Additionally, we obtained 87.03 Gb (~188×) of short-read Illumina 2 × 150 bp paired-end sequence data and combined it with long-read data to error correct and produce a hybrid assembly of 490.8 Mb. This assembly, AmGA_v0.1, consisted of 1004 scaffolds with an N50 of 3.12 Mb. To further improve the assembly, we obtained 264.58 Gb (~561×) of Bionano optical mapping data (Supplementary Table 1) and combined it with AmGA_v0.1. Numerous corrections and 69 conflict cuts were made to AmGA_v0.1 using optical mapping. The improved version, AmGA_v0.2, had significantly improved assembly made of just 88 scaffolds with an N50 of 7.4 Mb. We combined the AmGA_v0.2 assembly with 39.9 Gb (~86×) of Hi-C chromosome conformation capture data consisting of ~51 million valid di-tags, which included 7.47 million, 22.55 million, and 20.98 million Cis-close, Cis-far, and Trans di-tags, respectively. The resulting assembly, AmGA_v0.3, included 291 contigs that were represented by 33 scaffolds corresponding to 31 chromosomes, a scaffold with organelle genomes, and an unplaced scaffold (Fig. 3b). Further, gap-filling and error corrections in AmGA_v0.3 generated a 456.6 Mb reference genome (AmGA_v1.0) containing 98.7% of the genome (Fig. 2 and Table 1). The genome features of *A. marina* compared with *Oryza sativa* and *Arabidopsis thaliana* are given in Table 2.

**Table 1:** *A. marina* genome assembly statistics.

Assembly	Contigs (*n*)	Scaffolds (*n*)	Gaps Mb (%)	Contig N50 (Mb)	Scaffold N50 (Mb)	Assembly Size (Mb)
AmGA_v0.1 (PacBio + Illumina)	1277	1004	0.04 (0.008)	1.77	3.12	490.8
AmGA_v0.2 (v0.1 + Bionano)	290	88	1.18 (0.26)	2.79	7.41	457.7
AmGA_v0.3 (v0.2 + Hi-C)	291	33	1.19 (0.26)	2.75	14.6	457.7
AmGA_v1.0 (v0.3 + Gap closing and error correction)	252	32	1.10 (0.24)	3.00	14.6	456.6

**Table 2:** Comparison of *A. marina* genome assembly (AmGA_v1.0) to other high-quality genomes.

Genome features	*Oryza sativa* (IRGSP1.0)	*Arabidopsis thaliana* (TAIR10)	*Avicennia marina* (current study)
Estimated genome size (Mb)	384.2	135.0	462.7
Number of chromosomes (*n*)	12	5	31
Total sequence length assembled (Mb)	382.8	134.6	456.6
Total assembly gap length (Mb)	9.6	0.15	1.1
Number of scaffolds	12	5	32
Scaffold N50 (Mb)	29.9	23.46	14.6
Longest Scaffold (Mb)	45.0	30.42	22.9

### Genome completeness and validation

The completeness of the *A. marina* genome was assessed by BUSCO analysis, and the genome was validated by mapping RNA-Seq data from multiple tissues. BUSCO analysis revealed 99% complete BUSCOs, which included 94% complete and single-copy BUSCOs, and 5% complete and duplicated BUSCOs. When RNA-Seq reads from roots, pneumatophores, leaves, flowers, and developing seeds were mapped to the *A. marina* genome, we observed that the mapped and uniquely mapped reads were 97% and 98%, respectively.

### Annotation of *A. marina* genome

The repetitive sequences in the *A. marina* genome were annotated and masked before annotating the protein-coding genes. Both *in silico* and homology-based approaches were used for this purpose. The *A. marina* genome contained 51.6% repetitive sequences, which predominantly included retroelements (20.97%), long terminal repeats (LTR) elements (20.38%), Gypsy/DIRS1 elements (10.09%), Ty1/Copia elements (9.70%), and DNA transposons (3.18%). Details of all the repeat elements in the *A. marina* genome are given in Supplementary Table 2 and Fig. 3a. With the repeat-masked genome, gene prediction was carried out using ab initio, homology-based, and evidence-based methods. From the consensus gene models, 31,477 genes with 2953 bp average gene size and 1146 bp average coding sequence length were predicted (Supplementary Table 3 and Supplementary Figure 2). Among the 31,477 protein-coding genes, we were able to assign putative functions for 24,917 genes. Of the remaining 4097 genes with unknown functions, 2463 had no significant similarity to known protein-coding genes. Based on gene ontology (GO) annotation, 18,616 genes were classified under biological processes, molecular functions, and cellular components (Supplementary Fig. 3). Genes related to “organic substance metabolic processes”, “heterocyclic compound binding”, and “membrane” were the most abundant in the biological processes, molecular functions, and cellular components, respectively. Transcription factors (TFs) belonging to 70 TF families were predicted from the *A. marina* genome. Genes coding for the bHLH TF family were the most abundant (178 genes), followed by MYB (151 genes), C2H2 (147 genes), and AP2 (133 genes) TF families (Supplementary Table 4).

### Synteny and duplication analysis

Genomic synteny analysis of *A. marina* genome using MCScanX revealed the presence of 2114 syntenic blocks across the genome, including 19,576 genes. The syntenic blocks have shown that 14,473 paralogous gene pairs and 5103 genes are connected inter-chromosomally and intra-chromosomally, respectively (Fig. 3a). MCScanX analysis revealed the type of duplications in the gene paralogs of *A. marina*. Most of the *A. marina* genes were classified under whole-genome duplications (WGDs) or segmental duplications (16,610 genes, 52.7%). The remaining genes were classified under dispersed (21.7%), proximal (3.4%), and tandem duplications (5.9%) or singletons (13.6%).

### Assembly of the organelle genomes

A complete circular chloroplast genome of 147.7 kb was assembled, which contained a 17.8 kb small single-copy (SSC) region and an 87.5 kb large single-copy (LSC) region. The repeat size of the inverted repeat (IR) region was 21.2 kb. Annotation of the chloroplast genome revealed 123 genes, including 81 coding sequences (CDS), 35 tRNA genes, and eight rRNA genes. We also assembled a complete circular mitochondrial genome with a genome size of 579 kb. The mitochondrial genome consisted of 103 genes, including 62 CDS, 35 tRNA genes, and six rRNA genes. The landscape of chloroplast and mitochondrial genomes are presented in Fig. 3c, d.

### The salinome of *A. marina*

**Fig. 4:**
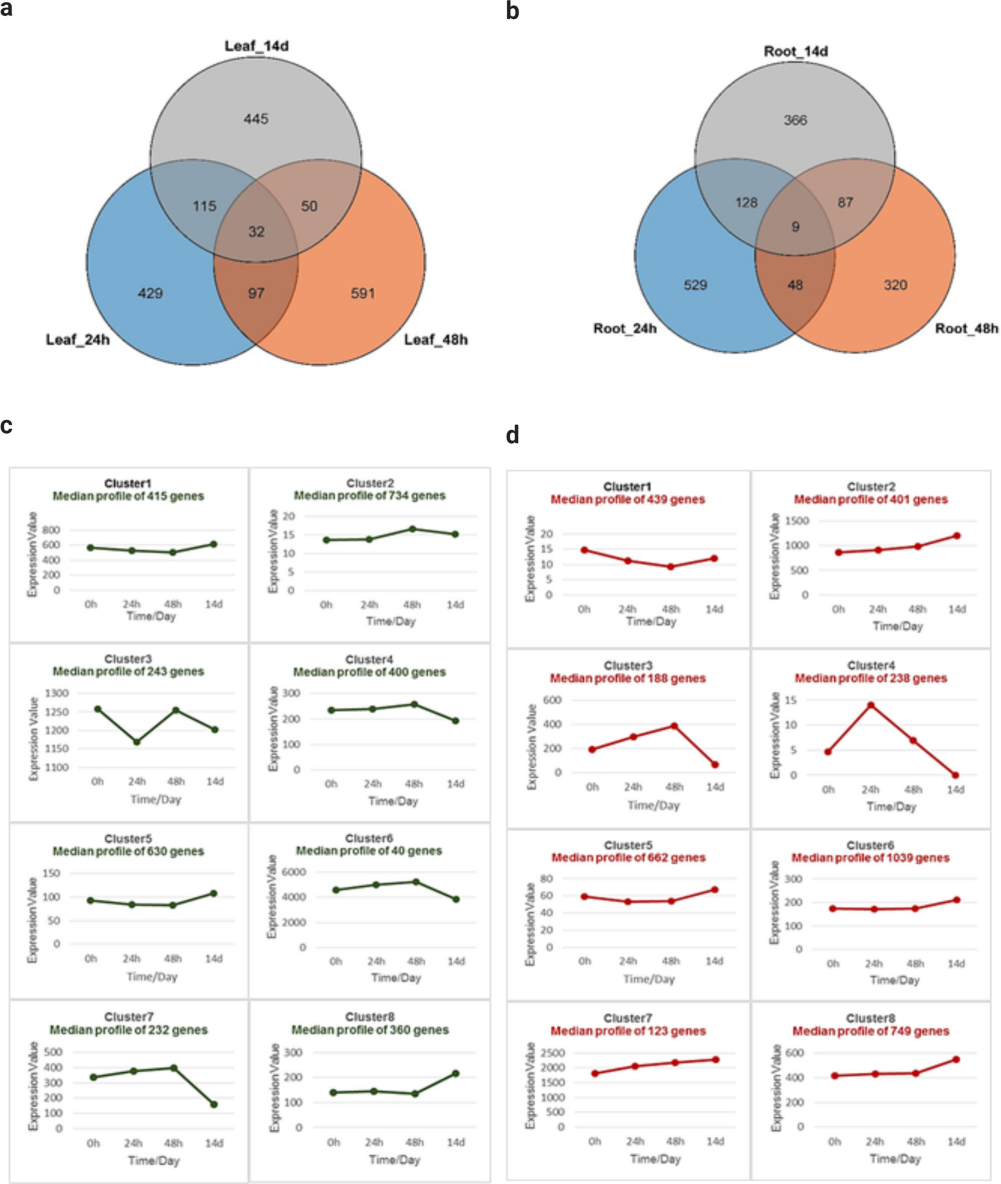
Salinity-responsive genes from *A. marina*. Leaf and root tissues were collected from control (Hoagland’s solution) and salt-treated (Hoagland’s solution supplemented with NaCl) seedlings and salinity-responsive genes were identified by RNA-Seq analysis. **a**, **b** Venn diagram of the differentially expressed genes (DEGs) from *A. marina* leaves and roots in response to short-term (24, 48 h) and long-term (14d) salinity stress that were analyzed in control-treatment pairs. **c**, **d** Time-course analysis and clustering of the DEGs based on their pattern of expression in leaves and roots, respectively.

We analyzed the RNA from the leaves and roots of *A. marina* seedlings hydroponically grown in Hoagland’s solution^27^ and supplemented with sodium chloride for short-term (24 h or 48 h) and long-term (14d) durations to identify the salinity-responsive genes. Seedlings continuously grown in Hoagland’s solution were used as control. About 10 million quality-filtered reads were mapped to the *A. marina* genome, and the differentially expressed genes (DEGs) in pair-wise combinations were identified. The DEGs were filtered with a minimum log2FC of 1 and FDR of 0.05. Differentially expressed gene (DEG) analysis identified 1759 genes in the leaves. The DEGs included 1203 that were upregulated and 556 that were downregulated. Between the treatments, 97 and 445 DEGs were exclusively present in the short-term and long-term salinity treatments, respectively. Similarly, 1487 genes were differentially expressed in the roots. Between the treatments, 46 and 366 DEGs were exclusively present in the short-term and long-term salinity treatments, respectively (Fig. 4). In the time-course analysis of gene expression, 3054 and 3849 DEGs (FDR < 0.05 and *R*-squared > 0.7) were identified in the leaves and roots, respectively. These DEGs were grouped into eight clusters based on their pattern of expression. DEGs in clusters 2, 4, 6, and 7 in the leaves, and 2, 3, 7, and 8 in the roots showed upregulated pattern in the short-term treatments. Similarly, DEGs in clusters 1, 5, and 8 in the leaves, and 1, 2, 5, 6, 7, and 8 in the roots showed upregulated pattern in the long-term treatment. DEGs in clusters 2, 7, and 8 showed upregulated pattern across all the treatments. Besides, we identified 614 genes homologous to the genes, which conferred salinity tolerance in transgenic plants. These genes were involved in various functions, including the oxidation–reduction process, removal of superoxide radicals, osmotic regulation, water transport, transmembrane ion transport, and signaling. A hypothetical pathway of salt secretion through the salt glands in *A. marina* and the genes involve are shown in Fig. 1c.

## Discussion

Mangrove plants thrive under high salinity with exposure to significant diurnal and seasonal variations in the salinity level. Understanding mangroves’ salinity tolerance mechanism at the molecular level is critical for developing crops that can be potentially cultivated using the abundant seawater. *A. marina* is one of the most salt-tolerant mangrove species bestowed with multiple salt-tolerance mechanisms, including salt-secreting glands in the leaves. It grows well in 75% seawater^28^ and tolerates a salinity equivalent to >250% seawater (92 ppt)^26^. Genome sequencing of *A. marina* will support the analysis of mangroves and other salt-tolerant plants to understand the evolution of salinity tolerance mechanisms in plants. *A. marina* is a diploid with 2*n* = 62 chromosomes^29^, and we estimated its genome size as 462.7 Mb. Recently, *A. marina* genome was de novo assembled without Bionano optical mapping data. The genome assembly consisted of 421 scaffolds and 674 contigs^10^. A large number of scaffolds and contigs implies significant gaps in the assembly. Lack of optical mapping data makes the assembly entirely dependent on sequencing data, limiting the ability to improve assembly quality. Reference-quality genome assemblies often incorporate optical mapping data to split the chimeric contigs that significantly increases the assembly quality and contiguity^30,31^. Orthogonal genome structure data from optical mapping enables independent and non-sequencing-based error corrections. We used the DNA and RNA isolated from a single plant and generated sequence data from Illumina, PacBio Sequel, Bionano DLS optical mapping, and Hi-C mapping technologies. We followed a sequential assembly strategy that combined the various data types to iterate and improve the genome. We assembled a 456.6 Mb of the estimated 462.7 Mb *A. marina* genome (98.7% genome coverage) in 31 chromosomes derived from 88 scaffolds and 252 contigs. The percentage of genomes in gaps was 0.26%. We observed ~99% completeness of the genome as measured by BUSCO analysis, and 98% uniquely mapped reads further validated the genome assembly. The features of the *A. marina* genome are comparable to those of the well-characterized monocot and dicot model species, rice, and *Arabidopsis*. Taken together, we can consider the *A. marina* genome assembled in this study as nearly complete and a reference-grade genome.

We used ab initio, homology-based, and evidence-based gene prediction methods and detected 31,477 protein-coding genes with an average length of 2953 bp. The average coding sequence (CDS) length of these genes (1146 bp) was comparable to that of the well-characterized *Arabidopsis* genome (1230 bp). The annotated genes of *A. marina* represented several genes and gene families that are related to salinity tolerance. Because of the hydrophobic barriers in the roots, water uptake in *A. marina* heavily relies on the symplastic pathway, facilitated by aquaporins^32,33^. The *A. marina* genome contained 56 aquaporin genes belonging to all five subfamilies. The majority belonged to tonoplast intrinsic proteins, followed by plasma membrane intrinsic proteins and nodulin26-like intrinsic proteins. We previously showed that monodehydroascorbate reductase (*MDAR*) from *A. marina* conferred salinity tolerance in transgenic tobacco plants^23^. The *A. marina* genome contained mitochondrial and peroxisomal *MDAR* genes, in addition to the *MDAR* gene located in the chloroplast genome. Dehydration responsive element binding (DREB) proteins or CRT element binding factors (CBFs), which belong to the APETALA2/ethylene-responsive factor (AP2/ERF) superfamily of transcription factors, activate several downstream genes and confer tolerance to high salinity^34,35^. We identified 18 genes in the *A. marina* genome that code for the DREB family of transcription factors. NAM, ATAF, and CUC (NAC) transcription factors, including the *AmNAC1* from *A. marina*, conferred salinity tolerance in transgenic tobacco, and yeast^25^. The *A. marina* genome contained 95 genes for NAC transcription factors, and 12 were induced upon salinity stress. MYB transcription factors play essential roles in stress responses, besides their association with plant growth and development, secondary metabolism, and signal transduction^36,37^. Previously, we showed that overexpression of the *AmMYB1* gene enhanced salinity tolerance in transgenic tobacco^22^. No other *MYB* genes were functionally characterized from mangroves thus far. In the present study, we annotated 74 *MYB* genes in the *A. marina* genome, and six were upregulated under salinity stress.

Our analysis shows that the salinome of *A. marina* consisted of 3246 genes responsive to salt stress. The time-course analysis revealed sets of genes that followed different expression patterns, which could be exploited in future studies related to short-term and long-term exposure to salinity. Detailed analysis of the DEGs revealed the absence of several genes reported to be salt-induced in glycophytes. This may be because some salt-induced genes in the glycophytes are constitutively expressed in the halophytes^38,39^. As a result, a comparison of gene expression between the control and salinity treated *A. marina* seedlings may not reveal the complete repertoire of salinity tolerance genes. While modulating gene expression is one way of adaptation, the mangrove might also have acquired coding variants and additional genes to evolve salinity tolerance. Accumulation of compatible solutes confer salinity tolerance in plants, and glycine betaine is the major compatible solute in *A. marina*^40,41^. Glycine betaine accumulating plants typically have one or two betaine aldehyde dehydrogenase (BADH) genes^42^, but *A. marina* has three genes. It also has a choline monooxygenase (CMO) gene (needed to convert choline to betaine aldehyde), which hitherto eluded cloning using conventional methods^41^. High-affinity K^+^ transporters (HKTs) unload Na^+^ from the xylem and protect the photosynthetic tissues from salt-induced damages^43^. *A. thaliana* has one HKT gene, but its halophyte relative *Thellungiella salsuginea* has three genes arranged in a tandem array^44^. Remarkably, *A. marina* genome analysis revealed further expansion of HKT genes. It has five HKT genes; three tandemly arranged in chromosome 19 and two more located in chromosomes 19 and 5. However, the number of genes coding for several other transporters, enzymes, and structural proteins reported to be involved in salinity tolerance were the same in *A. marina* and other species. These results indicate that only specific genes needed to thrive under the saline environment are selectively expanded in *A. marina*. It is relevant to note that about 53% of the *A. marina* genes are classified under whole-genome duplications or segmental duplications. Functional studies are required to identify the enzymes, transmembrane proteins, and transcription factors that contribute individually and in a coordinated pathway to salinity tolerance. The hypothetical pathway of salt secretion exemplifies the complexity of the structures and the process involved in salinity tolerance.

Despite that over 1000 research publications have reported salt-tolerant transgenic plants, genetic modification of crop varieties suitable for cultivation in saline soils remains a significant challenge. A contributing factor to this challenge is that more than 90% of those genes functionally validated for salinity tolerance in the transgenic plants were cloned from glycophytes. Although comparative studies are limited, some genes from salt-tolerant halophytes have shown functional superiority, different regulation, and enhanced salinity tolerance compared to their homologs from salt-sensitive glycophytes^41,45–49^. The salt exclusion mechanisms of certain halophytic mangrove roots are much more efficient to the extent of developing bio-inspired seawater desalination technology than the sodium unloading mechanism of the glycophyte roots^50,51^. Further, there are about 65 halophytes that secrete the absorbed salts outside the plant system using salt glands, which are entirely lacking in glycophytes^52^. In this study, we identified 614 genes, including 159 transcription factors, which are homologous to the genes that were functionally validated for salinity tolerance in transgenic systems. These genes are predicted to be functionally superior, thus critical for genetic engineering of salinity tolerance. Additionally, analysis of their promoters could add further value to the genetic engineering strategies. Although salinity tolerance is a complex trait for genetic manipulations by transgenic approaches, it has been demonstrated that single gene transfers were sufficient to create even anatomical and morphological alterations such as succulent leaves, leaves with more trichomes, and roots with physical barriers to apoplastic salt entry^53–56^. Considering that unicellular trichomes and multicellular glands derived from trichome-like cells can secrete salts, genetically engineered trichomes in the above-described studies provide the hope that salt glands can be engineered by manipulating the existing genes and cell structures. Engineering salt glands, physical barriers, and ultrafiltration mechanisms, which help retain the salts outside the plant system, would be critically needed to achieve a breakthrough in breeding salt-tolerant crops. The *A. marina* genome presented in this study should serve as a crucial resource to accomplish this challenging task.

## Methods

### Plant material

Plant samples for this study were collected from Pichavaram Mangrove Forest, Tamil Nadu, India. A healthy *A. marina* tree was identified and tagged with an accession number, PICH2015. Tissues collected from *A. marina* PICH2015 were used for genome sequencing and transcriptome sequencing. Seedlings raised from the seeds of *A. marina* PICH2015 were used for the identification of the salinity-responsive genes.

### Illumina paired-end sequencing

Genomic DNA was extracted from the young leaf tissue of *A. marina* PICH2015 using DNeasy Plant Maxi Kit (Qiagen, Germany). Illumina DNA sequencing library was prepared using the TruSeq DNA PCR-Free Library Preparation Kit as per the manufacturer’s protocol (Illumina, USA). Genomic DNA (1 µg) was fragmented to an average insert size of 350 bp using an ultrasonicator (Covaris, USA). The overhangs in the fragmented DNAs to blunt ends using the end-repair mix. The 3′ ends of the blunt-ended fragments were adenylated to prevent re-ligation of the fragments. The Ilumina index adapters were ligated to the DNA fragments and amplified using Illumina sequencing primers to generate a genomic DNA library. The library was purified, quantified using Qubit Fluorometer (Invitrogen, USA), and assessed for its quality using 2100 Bioanalyzer (Agilent, USA). The library was sequenced using mid-output flow cells with paired-end sequencing chemistry using the NextSeq 500 platform (Illumina, USA).

### PacBio single-molecule real-time (SMRT) sequencing

Genomic DNA was isolated from the young leaves of *A. marina* PICH2015, a standard SMRTbell library was prepared using 50 µg of DNA and SMRTbell Template Prep Kit 1.0, according to the manufacturer’s recommendations (Pacific Biosciences, CA, USA). Size-selection of the library was carried out using the BluePippin size-selection system (Sage Science, MA, USA) to enrich the fragments of 20 kb size. The size-selected library was purified with AMPure PB beads. The library was assessed using Qubit Fluorometer (Invitrogen, USA) and FEMTO Pulse System (Agilent, USA). The DNA/Polymerase Binding Kit P6 was used for binding the DNA templates of the library to the DNA polymerase P6. SMRT sequencing of the library was done with SMRT cells (SMRT 1M v3 LR) and the Sequel DNA Sequencing Kit 3.0 with P6-C4 chemistry in the PacBio Sequel System (Pacific Biosciences, CA, USA).

### Bionano optical mapping

Healthy shoot tips with emerging leaf buds in *A. marina* PICH2015 were covered with black-colored paper bags for in planta etiolation to reduce the chlorophyll content. After seven days of in planta etiolation, 30–45 cm long twigs containing the shoots were cut and immediately immersed in the water collected from the mangrove site in a container. The twigs were maintained under dark condition and transported to the laboratory the same day. The partially etiolated and pale green-colored youngest leaves from the shoot tips were freshly harvested and used for DNA isolation. High-molecular-weight genomic DNA was isolated using Bionano Prep Plant DNA Isolation Kit by following a protocol for the plant tissues with high polyphenol content as recommended by the manufacturer (Bionano Genomics, USA). The DNA was assessed for its quality using the NanoDrop Spectrophotometer and FEMTO Pulse System (Agilent, USA), and quantity using the Qubit Fluorometer (ThermoFisher, USA). About 750 ng of genomic DNA was fluorescently labeled using the DLE-1 enzyme and DLS DNA Labeling Kit according to the manufacturer’s recommendations (Bionano Genomics, USA). The fluorescently labeled DNA fragments were loaded onto a Saphyr chip. The DNA fragments were linearized in the nanochannel arrays of the chip and imaged using the BioNano Genomics Saphyr System (Bionano Genomics, USA).

### Dovetail Hi-C sequencing

Young leaves were harvested from *A. marina*, and the Dovetail Hi-C Kit as per the manufacturer’s recommendations (Dovetail Genomics, IL, USA) was used for preparing a Hi-C library. About 250 mg of leaf tissue was used for crosslinking, and chromatin preparation using phosphate-buffered saline and formaldehyde. The sample was added to the chromatin capture beads and digested with *Dpn*II restriction enzyme, followed by end-filling to convert the sticky ends to blunt ends. The DNA was subjected to intra-aggregate DNA end ligation, and the crosslinks were reversed by proteinase K treatment. The DNA was purified using magnetic beads and fragmented to an average insert size of 350 bp. The size-selected DNA was used for library construction and sequenced using a mid-output flow cell with paired-end sequencing chemistry in the NextSeq 500 platform (Illumina, USA).

### Genome size estimation

For the estimation of genome size, canonical k-mers from the 2 × 150 bp Illumina paired-end sequencing data were counted using Jellyfish v. 2.3.0^57^, and k-mer frequency distribution for 17, 21, 25, and 31-mers were obtained. A histogram generated and analyzed in GenomeScope (http://qb.cshl.edu/genomescope/). The genome size and heterozygosity percentage were estimated based on a k-mer-based statistical approach.

### Assembly of *A. marina* genome

The sequencing adapters and low-quality reads (Phred score QV < 30) from the 2 × 150 bp Illumina paired-end sequencing reads were removed using the Trimmomatic tool^58^. PacBio sequencing reads were error corrected using Canu assembler^59^. The quality-filtered reads from Illumina and Pacbio sequencing were combined and used for the hybrid genome assembly using MaSuRCA genome assembler with default parameters^60^. The hybrid assembly was combined with Bionano optical mapping data for conflict resolution using BioNano Solve tools (Bionano Genomics, USA). To further improve the assembly, the Hi-C data from *A. marina* was used after removing the sequencing adapters and low-quality reads (Phred score QV < 30) using the Trimmomatic tool^58^. The scaffolds from the hybrid assembly were combined with Hi-C data for chromosome-scale de novo assembly using the HiRise scaffolding tool (Dovetail Genomics, USA). The gaps in the chromosome-scale assembly were closed using TGS-GapCloser (https://github.com/BGI-Qingdao/TGS-GapCloser) and subjected to a final error correction using the Illumina paired-end sequencing data and Pilon tool (https://github.com/broadinstitute/pilon/).

### Assembly of *A. marina* organelle genomes

One scaffold (815.2 kb), which did not cluster with the other chromosomes of *A. marina*, was separated and used as a source for the assembly of organelle genomes. This scaffold contained three contigs of 189.9, 409.4, and 215.8 kb. The 189.9 kb contig showed similarity to the chloroplast genomes, and therefore, the paired-end reads of this contig were used to assemble the chloroplast genome using NOVOPlasty (https://github.com/ndierckx/NOVOPlasty). The other two contigs, which showed similarity to the mitochondrial genomes, were used to assemble the mitochondrial genome using Geneious Prime (https://www.geneious.com/prime/). The chloroplast and mitochondrial genomes of *A. marina* were annotated using the GeSeq tool, and their circular structures were created using OGDRAW (https://chlorobox.mpimp-golm.mpg.de/geseq.html).

### Annotation of *A. marina* genome

A de novo repeat library was constructed for the *A. marina* genome using the Repeat-Modeler tool (http://www.repeatmasker.org/RepeatModeler/), and the repetitive sequences were annotated based on the PGSB repeat element database (http://pgsb.helmholtz-muenchen.de/plant/plantsdb.jsp) and Repbase (https://www.girinst.org/repbase/) using RepeatMasker tool (http://www.repeatmasker.org/). The repeat-masked sequences were used for genome annotation. The genome was annotated using MAKER tool^61^ and EVidence Modeler tool^62^ by combining ab initio, homology-based, and evidence-based gene prediction methods. De novo ab initio gene predictions were performed using AUGUSTUS^63^ and SNAP^64^. AUGUSTUS was self-trained with the full-length transcripts using MAKER. *Arabidopsis thaliana* was set as the training organism for both AUGUSTUS and SNAP ab initio gene predictions. Homology-based gene predictions were carried out with the protein sequences from *Sesamum indicum, Erythranthe guttata, Olea europaea, Hevea brasiliensis*, and *Gossypium hirsutum*, which were closely related to *A. marina*. For evidence-based gene predictions, Illumina RNA-Seq data and PacBio Iso-Seq data from roots, leaves, flowers, developing seeds, and pneumatophores of *A. marina* were used. The RNA-Seq reads were de novo assembled to 304,905 transcripts using Trinity^65^. The Iso-Seq reads were de novo assembled to 34,094 full-length transcripts using SMRT Analysis 2.3 tools (Pacific Biosciences, USA). The transcripts from RNA-Seq and Iso-Seq data were combined and clustered using the CD-HIT-EST tool (http://weizhongli-lab.org/cd-hit/), which yielded 336,206 transcripts. These transcript sequences served as a transcripts data set for the evidence-based gene predictions. EVidence Modeler tool was used, and a consensus gene set was built after combining the gene models from the three gene prediction methods. The predicted genes were assigned with putative functions based on homology search against RefSeq, InterPro, GO, and KEGG protein/functions databases using BLAST2GO^66^. Transcription factor families were predicted using the plant transcription factor database and iTAK tool (http://itak.feilab.net/cgi-bin/itak/online_itak.cgi).

### Analysis of the completeness of *A. marina* genome

The completeness of the *A. marina* genome assembly was tested against the Viridiplantae datasets Odb10 using BUSCO.v3 tool^67^. RNA-Seq reads from roots, leaves, flowers, developing seeds, and pneumatophores were mapped using STAR aligner^68^ and BWA aligner^69^, and the quality of the genome was assessed.

### Synteny analysis of *A. marina* genome

All-vs-all BLASTP searches were done, and the paralogous or orthologous gene pairs were identified with 10^−5^ *E-*value cutoff. The collinear blocks were identified using MCScanX^70^ and TBtools (https://github.com/CJ-Chen/TBtools). A circos structure was developed using CIRCA tool (http://omgenomics.com/circa/).

### Transcriptome sequencing by PacBio Iso-seq

Total RNA was isolated from roots, leaves, flowers, developing seeds, and pneumatophores of *A. marina* using RNeasy Plant Mini Kit (Qiagen, Germany). The RNA was purified after DNase I treatment using the RNeasy MinElute Cleanup Kit (Qiagen, Germany). The quality and quantity of the RNA were assessed using a spectrophotometer, Qubit Fluorometer (ThermoFisher, USA), and 2100 Bioanalyzer (Agilent, USA). SMARTer PCR cDNA Synthesis Kit was used, and cDNA was prepared as per the manufacturer’s protocol (Takara, USA). SMRTbell library was prepared using SMRTbell Template Prep Kit 1.0 (Pacific Biosciences, CA, USA), and size-selected using the BluePippin size-selection system (Sage Science, MA, USA). The size-selected library was purified and assessed using Qubit Fluorometer (Invitrogen, USA) and FEMTO Pulse System (Agilent, USA). The DNA/Polymerase Binding Kit P6 was used for the binding of the DNA polymerase P6 to the cDNA templates in the SMRTbell library. The polymerase-bound SMRTbell library was subjected to single-molecule real-time (SMRT) sequencing using SMRT cells (SMRT 1M v3 LR) and Sequel DNA Sequencing Kit 3.0 with P6-C4 chemistry in PacBio Sequel System (Pacific Biosciences, CA, USA).

### Illumina RNA-Seq

Total RNA was isolated from roots, leaves, flowers, developing seeds, and pneumatophores of *A. marina* using RNeasy Plant Mini Kit. The RNA was purified after DNase I treatment using the RNeasy MinElute Cleanup Kit (Qiagen, Germany). The quality and quantity of the RNA were assessed using a spectrophotometer, Qubit Fluorometer (ThermoFisher, USA), and 2100 Bioanalyzer (Agilent, USA). Separate RNA-Seq libraries were prepared for the RNA from different tissues using NEBNext Ultra II RNA Library Prep Kit (NEB, USA). Messenger RNA was purified from total RNA using Oligo-dT magnetic beads and fragmented with fragmentation buffer. Random hexamers were used to synthesize double-strand cDNAs from the fragmented mRNAs. The cDNAs were blunt-ended by end-repair, and sequencing adapters were ligated. The library was enriched by amplification using the sequencing primers and assessed for its insert size using 2100 Bioanalyzer (Agilent, USA). The library was quantified using Qubit Fluorometer (Invitrogen, USA), diluted to 4.0 nM concentration, and denatured using sodium hydroxide. The libraries were sequenced with paired-end (2 × 150 bp) sequencing chemistry using the NextSeq 500 platform (Illumina, USA).

### Identification of salinity-responsive genes

Seeds were collected from *A. marina* PICH2015, germinated, and grown for 1 week in sand beds. The one-week-old seedlings were transferred to the hydroponics system and grown in Hoagland’s solution^27^. One-month-old seedlings of the same height and vigor were transferred to the Hoagland’s solution supplemented with NaCl for salinity treatment. Short-term salinity treatment was given with 500 mM NaCl for 24 and 48 h and long-term salinity treatment was provided with 250 mM NaCl for 14 days. Seedlings grown in Hoagland’s solution were used as control. Three replicates were maintained for all the treatments and control. Leaves and roots were collected from the control and NaCl-treated seedling for whole transcriptome gene expression studies.

RNA-Seq libraries were prepared as described above (under Illumina RNA-Seq). The library from each sample was sequenced with paired-end (2 × 75 bp) sequencing chemistry using the NextSeq 500 platform (Illumina, USA). The reads were quality-filtered and mapped to the *A. marina* genome using the STAR RNA-Seq aligner tool^68^. BAM alignment, general feature format (GFF), and HTSeq R package (https://htseq.readthedocs.io/en/master/) were used, and a read count table was created for the genes across all the samples. The differentially expressed genes (DEGs) in different experimental pair-wise combinations were identified using edgeR package^71^ with individually calculated dispersion value and counts per million (CPM) of 1. The DEGs with log2FC < 1 and false discovery rate (FDR) < 0.05 were filtered out. Next, maSigPro R package with maSigPro model-based clustering method^72^ was used for time-course gene expression analysis. DEGs in the clusters were filtered based on FDR < 0.05 and *R*-squared > 0.7. The DEGs were annotated using BLAST2GO^66^.

### Identification of the homologs of experimentally validated salinity tolerance genes

We found 1030 publications from Pubmed in which one or more genes from plants were experimentally validated for salinity tolerance using transgenic approaches (last accessed 15th October 2020). We obtained the nucleotide sequence of these genes from Genbank or directly from the authors and performed a BLAST search to identify the homologous genes in *A. marina* (Supplementary Data 1).

### Reporting summary

Further information on research design is available in the Nature Research Reporting Summary linked to this article.

## Supplementary information

Supplementary Information

Description of Supplementary Files

Supplementary Data 1

Supplementary Data 2

Reporting summary

## Data Availability

The data reported in this study are available at DDBJ/ENA/GenBank (JACDXK000000000), NCBI (PRJNA392013), and SRA databases (PRJNA392014, PRJNA643813, and PRJNA644122).
