# Supplementary material for: A reference-grade genome identifies salt-tolerance genes from the salt-secreting mangrove species Avicennia marina
Source: Commun Biol. 2021 Jul 8;4:851. doi: 10.1038/s42003-021-02384-8 (PMC8266904; doi:10.1038/s42003-021-02384-8)
Supplement: Supplementary file 1 — Supplementary Information [file 42003_2021_2384_MOESM1_ESM.pdf]

**Supplementary Information**

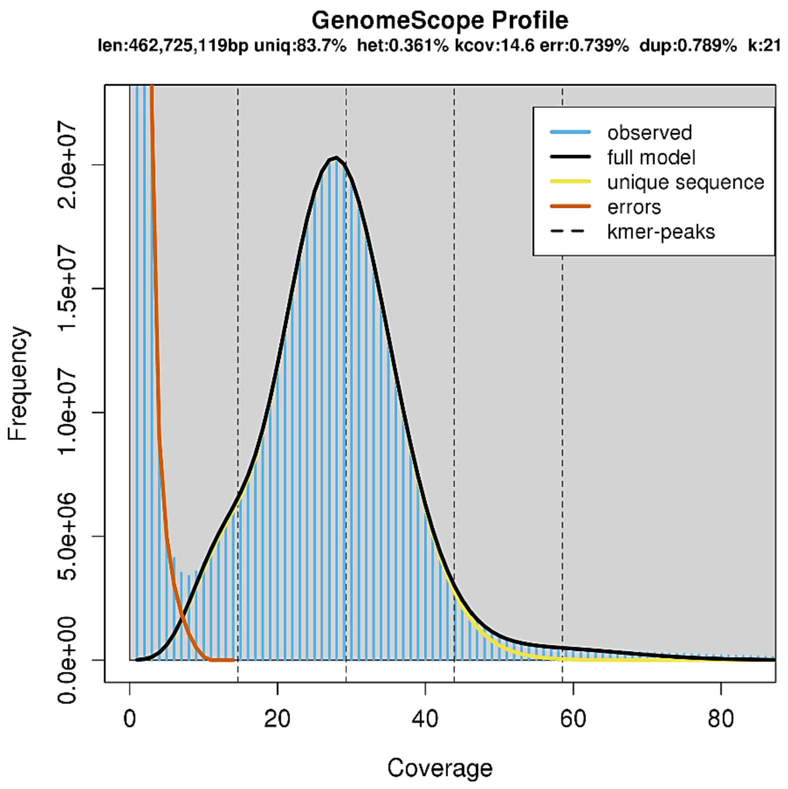


**Supplementary Figure 1 |** Estimation of *A. marina* genome size and heterozygosity by k-mer analysis.

**Supplementary Table 1 |** Molecule quality report of Bionano data from *A.marina*

| **Particulars** | **Data** |
|---|---|
| Total DNA (>= 20 kb) | 430.2466 Gb |
| N50 (>= 20 kb) | 0.1114 Mb |
| Total DNA (>=150 kb) | 142.9727 Gb |
| N50 (>=150 kb) | 0.2059 Mb |
| Total DNA (>= 150 kb & minSites >= 9) | 98.7988 Gb |

| | |
|---|---|
| N50 (>=150 kb & minSites >= 9) | 0.2111 Mb |
| Enzyme | DLE-1 |
| Label color | Green_01 |
| Average label density (>= 150 kb) | 8.34/100 kb |

**Supplementary Table 2 |** Repetitive sequences annotated in the *A. marina* genome.

| Particulars | Number of elements* | Length occupied (bp) | Percentage of genome |
|---|---|---|---|
| Retroelements | 88800 | 95735680 | 20.97 |
| Penelope | 401 | 102697 | 0.02 |
| LINEs | 2888 | 2665482 | 0.58 |
| L1/CIN4 | 2487 | 2562785 | 0.56 |
| LTR elements: | 85912 | 93070198 | 20.38 |
| Ty1/Copia | 41700 | 44299353 | 9.70 |
| Gypsy/DIRS1 | 40879 | 46086861 | 10.09 |
| Retroviral | 121 | 57279 | 0.01 |
| DNA transposons | 17935 | 14515603 | 3.18 |
| hobo-Activator | 3104 | 2609347 | 0.57 |
| Tourist/Harbinger | 1768 | 1201981 | 0.26 |
| Rolling-circles | 686 | 478813 | 0.10 |
| Unclassified | 389126 | 119349514 | 26.14 |
| Total interspersed repeats | | 229600797 | 50.28 |
| Small RNA | 296 | 58438 | 0.01 |

| | | | |
|---|---|---|---|
| Satellites | 1480 | 615574 | 0.13 |
| Simple repeats | 106389 | 3931467 | 0.86 |
| Low complexity | 18140 | 873868 | 0.19 |
| Total bases | | 235558957 | 51.59 |

* most repeats fragmented by insertions or deletions have been counted as one element

**Supplementary Table 3 |** Detailed information on the genes annotated from the *A. marina* genome and their comparison with *A. thaliana* and *O. sativa*

| **Genome features** | ***A. marina*** | ***A. thaliana*** | ***O. sativa*** |
|---|---|---|---|
| Number of genes | 31477 | 27416 | 39049 |
| Number of mRNAs | 31477 | 27416 | 39049 |
| Number of CDSs | 31477 | 27416 | 39049 |
| Number of exons | 160193 | 145520 | 169100 |
| Number of exons in CDS | 160193 | 140592 | 160725 |
| Number of introns in CDS | 128716 | 113176 | 121676 |
| Number of introns in exon delete? | 128716 | 118104 | 130051 |
| Number of single exon gene | 6845 | 6122 | 9732 |
| Number of single exon mRNA | 6845 | 6122 | 9732 |
| Mean mRNAs per gene | 1 | 1 | 1 |
| Mean CDSs per mRNA | 1 | 1 | 1 |
| Mean exons per mRNA | 5.1 | 5.3 | 4.3 |
| Mean exons per CDS | 5.1 | 5.1 | 4.1 |

| | | | |
|---|---|---|---|
| Mean introns in CDS per mRNA | 4.1 | 4.1 | 3.1 |
| Mean introns in exons per mRNA | 4.1 | 4.3 | 3.3 |
| Total gene length (bp) | 92973273 | 60480140 | 1.11E+08 |
| Total mRNA length (bp) | 92973273 | 59985740 | 1.1E+08 |
| Total CDS length (bp) | 36087135 | 33403693 | 41551661 |
| Total exon length (bp) | 36087135 | 40631639 | 55545366 |
| Total intron length per CDS (bp) | 57014854 | 17976713 | 49567983 |
| Total intron length per exon (bp) | 57014854 | 19472205 | 54758075 |
| Mean gene length (bp) | 2953 | 2206 | 2852 |
| Mean mRNA length (bp) | 2953 | 2187 | 2821 |
| Mean CDS length (bp) | 1146 | 1218 | 1064 |
| Mean exon length (bp) | 225 | 279 | 328 |
| Mean CDS piece length (bp) | 225 | 237 | 258 |
| Mean intron in CDS length (bp) | 442 | 158 | 407 |
| Mean intron in exon length (bp) | 442 | 164 | 421 |
| Longest gene (bp) | 63740 | 31258 | 57094 |
| Longest mRNA (bp) | 63740 | 31258 | 35678 |
| Longest CDS (bp) | 17667 | 16182 | 16311 |
| Longest exons (bp) | 7884 | 7761 | 15363 |
| Longest CDS piece (bp) | 7884 | 7761 | 15363 |
| Longest intron into CDS part (bp) | 39010 | 10235 | 18270 |
| Longest intron into exon part | 39010 | 10235 | 18270 |
| Shortest genes | 150 | 21 | 84 |

| | | | |
|---|---|---|---|
| Shortest mRNA | 150 | 21 | 84 |
| Shortest CDS | 150 | 21 | 18 |
| Shortest exons | 3 | 2 | 3 |
| Shortest CDS piece | 3 | 1 | 1 |
| Shortest intron into CDS part | 25 | 9 | 6 |
| Shortest intron into exon part | 25 | 9 | 6 |
| Number of mrnas with utr both sides | 12638 | 18847 | 20725 |
| Number of mrnas with at least one utr | 19304 | 21079 | 23476 |
| Number of five_prime_utrs | 15765 | 19537 | 21560 |
| Number of three_prime_utrs | 16177 | 20389 | 22641 |
| Number of exon in five_prime_utr | 22621 | 23264 | 26449 |
| Number of exon in three_prime_utr | 22798 | 21479 | 25985 |
| Number of intron in five_prime_utr | 6856 | 3727 | 4889 |
| Number of intron in three_prime_utr | 6621 | 1090 | 3344 |
| mean five_prime_utrs per mrna | 0.7 | 0.7 | 0.6 |
| mean three_prime_utrs per mrna | 0.8 | 0.7 | 0.6 |
| mean exons per five_prime_utr | 1.4 | 1.2 | 1.2 |
| mean exons per three_prime_utr | 1.4 | 1.1 | 1.1 |
| mean introns in five_prime_utrs per mrna | 0.3 | 0.1 | 0.1 |
| mean introns in three_prime_utrs per mrna | 0.3 | 0 | 0.1 |
| Total five_prime_utr length | 5306901 | 2671928 | 4575504 |
| Total three_prime_utr length | 9760638 | 4556018 | 9418201 |
| Total intron length per five_prime_utr | 3690708 | 1230380 | 3298168 |

| | | | |
|---|---|---|---|
| Total intron length per three_prime_utr | 3161816 | 231416 | 1803770 |
| mean five_prime_utr length | 336 | 136 | 212 |
| mean three_prime_utr length | 603 | 223 | 415 |
| mean five_prime_utr piece length | 234 | 114 | 172 |
| mean three_prime_utr piece length | 428 | 212 | 362 |
| mean intron in five_prime_utr length | 538 | 330 | 674 |
| mean intron in three_prime_utr length | 477 | 212 | 539 |
| Longest five_prime_utrs | 10135 | 2331 | 5329 |
| Longest three_prime_utrs | 9759 | 3118 | 6072 |
| Longest five_prime_utr piece | 8562 | 2331 | 5329 |
| Longest three_prime_utr piece | 9759 | 3118 | 5809 |
| Longest intron into five_prime_utr part | 9728 | 9725 | 17812 |
| Longest intron into three_prime_utr part | 11184 | 4454 | 17596 |
| Shortest five_prime_utrs | 1 | 1 | 1 |
| Shortest three_prime_utrs | 1 | 1 | 1 |
| Shortest five_prime_utr piece | 1 | 1 | 1 |
| Shortest three_prime_utr piece | 1 | 1 | 1 |
| Shortest intron into five_prime_utr part | 11 | 21 | 12 |
| Shortest intron into three_prime_utr part | 21 | 33 | 35 |

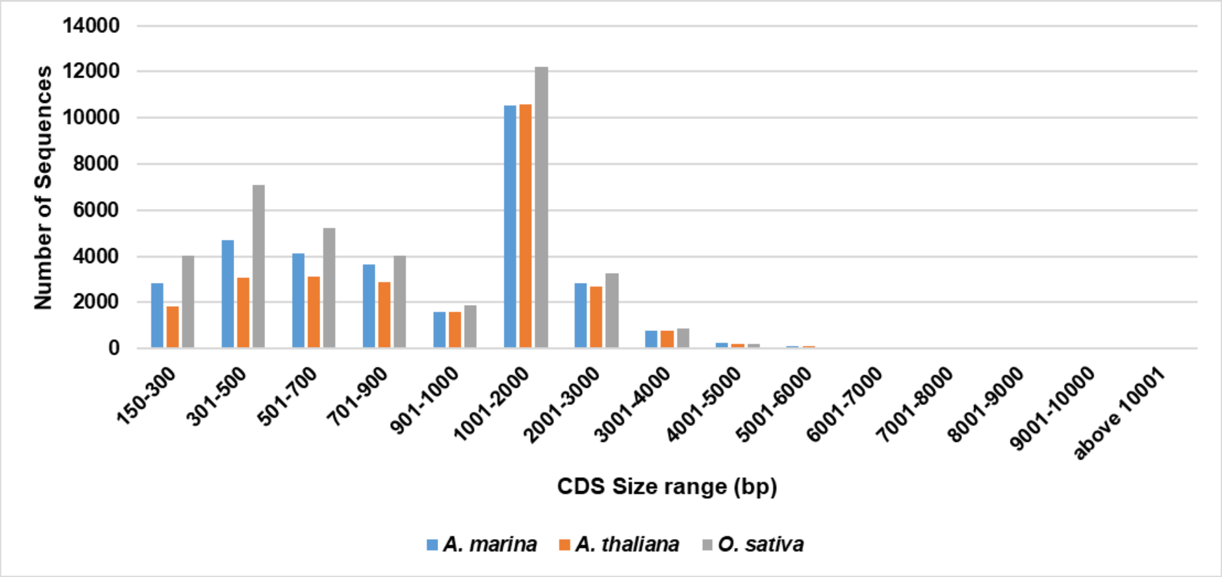


**Supplementary Figure 2 |** Size distribution of the coding sequences predicted from the *A. marina* genome and their comparison with *A. thaliana* and *O. sativa*.

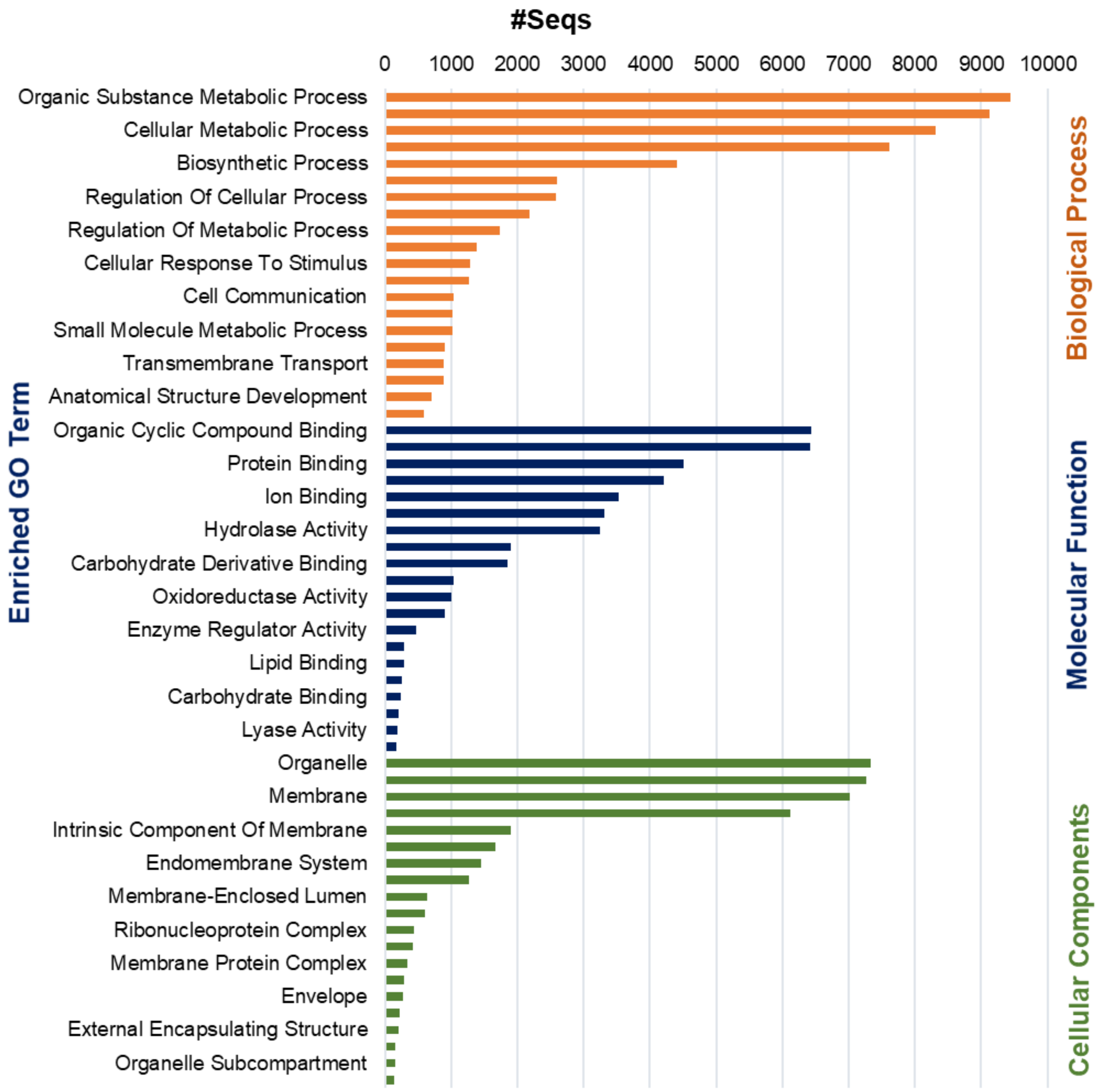


**Supplementary Figure 3 |** Gene ontology (GO) category distribution among the 18,616 genes annotated under the GO terms biological process, molecular function, and cellular components.

**Supplementary Table 4 |** The number of genes coding the transcription factors predicted from the *A. marina* genome

| Transcription Factor Family | Number of genes |
|---|---|
| bHLH | 178 |
| MYB-related | 151 |
| C2H2 | 147 |
| AP2/ERF-ERF | 133 |
| bZIP | 101 |
| WRKY | 100 |
| NAC | 95 |
| MADS-M-type | 77 |
| MYB | 74 |
| GRAS | 62 |
| C3H | 61 |
| C2C2-Dof | 61 |
| HB-HD-ZIP | 55 |
| GARP-G2-like | 55 |
| Trihelix | 48 |
| TCP | 46 |
| LOB | 44 |
| B3 | 42 |
| C2C2-GATA | 39 |

| | |
|---|---|
| HSF | 38 |
| B3-ARF | 35 |
| OFP | 34 |
| FAR1 | 27 |
| SBP | 23 |
| HB-BELL | 22 |
| HB-WOX | 21 |
| NF-YB | 21 |
| Tify | 19 |
| HB-other | 18 |
| TUB | 18 |
| C2C2-CO-like | 17 |
| RWP-RK | 15 |
| GeBP | 15 |
| AP2/ERF-AP2 | 14 |
| MADS-MIKC | 14 |
| GARP-ARR-B | 13 |
| NF-YA | 12 |
| DDT | 12 |
| PLATZ | 12 |
| SRS | 12 |
| LIM | 11 |
| BES1 | 11 |

| | |
|---|---|
| C2C2-YABBY | 11 |
| Alfin-like | 10 |
| NF-YC | 8 |
| zf-HD | 8 |
| BBR-BPC | 7 |
| CPP | 7 |
| EIL | 6 |
| CAMTA | 6 |
| LFY | 4 |
| C2C2-LSD | 4 |
| DBB | 4 |
| AP2/ERF-RAV | 4 |
| E2F-DP | 4 |
| S1Fa-like | 3 |
| VOZ | 3 |
| DBP | 3 |
| NOZZLE | 2 |
| SAP | 2 |
| Whirly | 2 |
| HB-PHD | 2 |
| NF-X1 | 2 |
| CSD | 1 |
| STAT | 1 |

| | |
|---|---|
| ULT | 1 |
| GRF | 1 |
| HB-KNOX | 1 |
| HRT | 1 |
| BSD | 1 |
