# Supplementary material for: A reference-grade genome identifies salt-tolerance genes from the salt-secreting mangrove species Avicennia marina
Source: Commun Biol. 2021 Jul 8;4:851. doi: 10.1038/s42003-021-02384-8 (PMC8266904; doi:10.1038/s42003-021-02384-8)
Supplement: Supplementary file 2 — Description of Supplementary Files [file 42003_2021_2384_MOESM2_ESM.pdf]

Description of Additional Supplementary Files

File name: Supplementary Data 1

Description: Details of experimentally validated genes for salt tolerance and their homolog from *A. marina* genome.

File name: Supplementary Data 2

Description: Source data for all graphs and charts.
