# Supplementary material for: A reference-grade genome identifies salt-tolerance genes from the salt-secreting mangrove species Avicennia marina
Source: Commun Biol. 2021 Jul 8;4:851. doi: 10.1038/s42003-021-02384-8 (PMC8266904; doi:10.1038/s42003-021-02384-8)
Supplement: Supplementary file 5 — Reporting summary [file 42003_2021_2384_MOESM5_ESM.pdf]

nature research

Corresponding author(s): Madasamy Parani

Last updated by author(s): Dec 16, 2020

Reporting Summary

Nature Research wishes to improve the reproducibility of the work that we publish. This form provides structure for consistency and transparency in reporting. For further information on Nature Research policies, see our Editorial Policies and the Editorial Policy Checklist.

Statistics

For all statistical analyses, confirm that the following items are present in the figure legend, table legend, main text, or Methods section.

n/a	Confirmed	
☐	☒	The exact sample size (*n*) for each experimental group/condition, given as a discrete number and unit of measurement
☐	☒	A statement on whether measurements were taken from distinct samples or whether the same sample was measured repeatedly
☐	☒	The statistical test(s) used AND whether they are one- or two-sided<br>*Only common tests should be described solely by name; describe more complex techniques in the Methods section.*
☒	☐	A description of all covariates tested
☐	☒	A description of any assumptions or corrections, such as tests of normality and adjustment for multiple comparisons
☐	☒	A full description of the statistical parameters including central tendency (e.g. means) or other basic estimates (e.g. regression coefficient) AND variation (e.g. standard deviation) or associated estimates of uncertainty (e.g. confidence intervals)
☐	☒	For null hypothesis testing, the test statistic (e.g. *F*, *t*, *r*) with confidence intervals, effect sizes, degrees of freedom and *P* value noted<br>*Give P values as exact values whenever suitable.*
☒	☐	For Bayesian analysis, information on the choice of priors and Markov chain Monte Carlo settings
☒	☐	For hierarchical and complex designs, identification of the appropriate level for tests and full reporting of outcomes
☒	☐	Estimates of effect sizes (e.g. Cohen's *d*, Pearson's *r*), indicating how they were calculated

Our web collection on statistics for biologists contains articles on many of the points above.

Software and code

Policy information about availability of computer code

Data collection	No commercial/custom code was used for data collection.
Data analysis	Here, we are listing the software URLs of all open-source /commercial tools used for data analysis.<br>R: https://www.R-project.org/<br>Jellyfish: https://www.cbcb.umd.edu/software/jellyfish/<br>GenomeScope: http://qb.cshl.edu/genomescope/<br>Trimmomatic: http://www.usadellab.org/cms/index.php?page=trimmomatic<br>Canu: https://github.com/marbl/canu<br>MaSuRCA:http://www.genome.umd.edu/masurca.html<br>BioNano Solve :https://bionanogenomics.com/downloads/bionano-solve/<br>HiRise scaffolding tool: https://github.com/DovetailGenomics/HiRise_July2015_GR<br>TGS-GapCloser:https://github.com/BGI-Qingdao/TGS-GapCloser<br>Pilon:https://github.com/broadinstitute/pilon/<br>NOVOPlasty:https://github.com/ndierckx/NOVOPlasty<br>OGDRAW:https://chlorobox.mpimp-golm.mpg.de/geseq.html<br>Geneious Prime :https://www.geneious.com/prime/<br>Repeatmasker: http://www.repeatmasker.org<br>Repeatmodeler: http://www.repeatmasker.org/RepeatModeler.html<br>MAKER: http://www.yandell-lab.org/software/maker.html<br>SNAP: http://snap.cs.berkeley.edu<br>AUGUSTUS: http://augustus.gobics.de/<br>Trinity:https://github.com/trinityrnaseq/trinityrnaseq/wiki

CD-HIT-EST tool: http://weizhongli-lab.org/cd-hit/
EVidence Modeler: https://evidencemodeler.github.io/
iTAK tool : http://itak.feilab.net/cgi-bin/itak/online_itak.cgi
BLAST2GO: https://www.blast2go.com/
BUSCO: https://gitlab.com/ezlab/busco
BWA: https://github.com/lh3/bwa/releases
TBtools: https://github.com/CJ-Chen/TBtools
CIRCA: http://omgenomics.com/circa/
MCScanX:http://chibba.pgml.uga.edu/mcscan2/
STAR: https://github.com/alexdobin/STAR
HTSeq: https://htseq.readthedocs.io/en/master/
EdgeR: https://bioconductor.org/packages/release/bioc/html/edgeR.html
Next maSigPro : https://bioconductor.org/packages/release/bioc/html/maSigPro.html

For manuscripts utilizing custom algorithms or software that are central to the research but not yet described in published literature, software must be made available to editors and reviewers. We strongly encourage code deposition in a community repository (e.g. GitHub). See the Nature Research guidelines for submitting code & software for further information.

Data

Policy information about availability of data

All manuscripts must include a data availability statement. This statement should provide the following information, where applicable:

- Accession codes, unique identifiers, or web links for publicly available datasets
- A list of figures that have associated raw data
- A description of any restrictions on data availability

The data reported in this study is publicly available at DDBJ/ENA/GenBank (JACDXK000000000), NCBI (PRJNA392013), and SRA databases (PRJNA392014, PRJNA643813, and PRJNA644122).

Field-specific reporting

Please select the one below that is the best fit for your research. If you are not sure, read the appropriate sections before making your selection.

[x] Life sciences [ ] Behavioural & social sciences [ ] Ecological, evolutionary & environmental sciences

For a reference copy of the document with all sections, see nature.com/documents/nr-reporting-summary-flat.pdf

Life sciences study design

All studies must disclose on these points even when the disclosure is negative.

Sample size	Leaf sample from a single plant Avicennia marina was used for the De novo genome sequencing.
Data exclusions	No data were excluded from the analysis.
Replication	Biological replicates were used as required and described in the methods section.
Randomization	Not applicable.
Blinding	Not applicable.

Reporting for specific materials, systems and methods

We require information from authors about some types of materials, experimental systems and methods used in many studies. Here, indicate whether each material, system or method listed is relevant to your study. If you are not sure if a list item applies to your research, read the appropriate section before selecting a response.

Materials & experimental systems

n/a	Involved in the study	
[x]	[ ]	Antibodies
[x]	[ ]	Eukaryotic cell lines
[x]	[ ]	Palaeontology and archaeology
[ ]	[x]	Animals and other organisms
[x]	[ ]	Human research participants
[x]	[ ]	Clinical data
[x]	[ ]	Dual use research of concern

Methods

n/a	Involved in the study	
[x]	[ ]	ChIP-seq
[x]	[ ]	Flow cytometry
[x]	[ ]	MRI-based neuroimaging

Animals and other organisms

Policy information about studies involving animals; ARRIVE guidelines recommended for reporting animal research

Laboratory animals	Not applicable.
Wild animals	Not applicable.
Field-collected samples	Plant samples for this study were collected from Pichavaram Mangrove Forest, Tamil Nadu, India. A healthy A. marina tree was identified and tagged with an accession number, PICH2015. Tissues collected from A. marina PICH2015 were used for genome sequencing and transcriptome sequencing. Seedlings raised from the seeds of A. marina PICH2015 were used for the identification of the salinity-responsive genes
Ethics oversight	Not applicable.

Note that full information on the approval of the study protocol must also be provided in the manuscript.
